# Growth-Dependent Catalase Localization in *Exiguobacterium oxidotolerans* T-2-2^T^ Reflected by Catalase Activity of Cells

**DOI:** 10.1371/journal.pone.0076862

**Published:** 2013-10-18

**Authors:** Yoshiko Hanaoka, Fumihiko Takebe, Yoshinobu Nodasaka, Isao Hara, Hidetoshi Matsuyama, Isao Yumoto

**Affiliations:** 1 Laboratory of Environmental Microbiology, Graduate School of Agriculture, Hokkaido University, Kita-ku, Sapporo, Japan; 2 Bioproduction Research Institute, National Institute of Advanced Industrial Science and Technology (AIST), Toyohira-ku, Sapporo, Japan; 3 School of Biological Science and Engineering, Tokai University, Minamisawa, Minami-ku, Sapporo, Japan; 4 Laboratory of Electron Microscopy, Graduate School of Dentistry, Hokkaido University, Kita-ku, Sapporo, Japan; University of Strathclyde, United Kingdom

## Abstract

A psychrotolerant and H_2_O_2_-resistant bacterium, *Exiguobacterium oxidotolerans* T-2-2^T^, exhibits extraordinary H_2_O_2_ resistance and produces catalase not only intracellularly but also extracellularly. The intracellular and extracellular catalases exhibited the same enzymatic characteristics, that is, they exhibited the temperature-dependent activity characteristic of a cold-adapted enzyme, their heat stabilities were similar to those of mesophilic enzymes and very high catalytic intensity. In addition, catalase gene analysis indicated that the bacterium possessed the sole clade 1 catalase gene corresponding to intracellular catalase. Hence, intracellular catalase is secreted into the extracellular space. In addition to intracellular and extracellular catalases, the inner circumference of the cells showed the localization of catalase in the mid-stationary growth phase, which was observed by immunoelectron microscopy using an antibody against the intracellular catalase of the strain. The cells demonstrated higher catalase activity in the mid-stationary growth phase than in the exponential growth phase. The catalase localized in the inner circumference can be dissociated by treatment with Tween 60. Thus, the localized catalase is not tightly bound to the inner circumference of the cells and may play a role in the oxidative defense of the cells under low metabolic state.

## Introduction

One of the reactive oxygen species (ROS), superoxide (O_2_ ˙^−^), is a byproduct of respiratory metabolism [1]–[4]. O_2_ ˙^−^ is decomposed to H_2_O_2_ by the reaction catalyzed by superoxide dismutase [5]. Although H_2_O_2_ itself is harmful to organisms, it produces a more harmful ROS, OH•, through the Fenton reaction [6]. Therefore, elimination of intracellular H_2_O_2_ by enzymes such as catalase, peroxidase or glutathione peroxidase is crucial for organisms to prevent ROS production, particularly OH•, which can damage to their cell components such as DNA, RNA, proteins and lipids [6]–[8].

The elimination of H_2_O_2_ is also important for not only aerobic microorganisms but also anaerobic microorganisms to survive in their own niches. For example, pathogenic, parasitic or symbiotic microorganisms interact with H_2_O_2_ produced by their host cells [9]–[11]. *Vibrio fisheri* eliminates H_2_O_2_ produced by the host squid in order to survive in the light organ of the host [11]. *V*. *fisheri* localizes catalase in the periplasmic space. On the other hand, *Vibrio rumoiensis*, which survives in drain water, in the presence of H_2_O_2_ produces catalase on its periplasmic space to decompose extracellular H_2_O_2_ as a defense mechanism [12], [13]. *V*. *rumoiensis* localizes catalase not only in the periplasmic space but also on the cell surface [14]. Thus, it appears that catalase expression and its localization in the cells of microorganisms are important for their defense against extracellular H_2_O_2_.

We have isolated extremophiles that have adapted to cold and oxidative (H_2_O_2_) environments to study bacterial and enzymatic adaptations to multiple extreme environments. Three novel species of psychrotolerant H_2_O_2_-resistant bacteria, namely, *V*. *rumoiensis* [12]–[17], *Exiguobacterium oxidotolerans* [18]–[20] and *Psychrobacter piscatorii* [21]–[23], have been isolated and their catalases have been studied. Among these isolates, *E. oxidotolerans* T-2-2^T^ is the only Gram-positive bacterium and it exhibits high catalase activity in the extracellular fraction. Gram-negative bacteria possess a periplasmic space between the inner and outer membranes. It has been reported that catalase exists in the periplasmic space as a defense mechanism against extracellular H_2_O_2_ [11], [24]. However, owing to the absence of the periplasmic space in Gram-positive bacteria, the defense mechanisms against extracellular H_2_O_2_ are not well understood. In the present study, the localization of catalase in the inner circumference of *E. oxidotolerans* T-2-2^T^ cells in the mid-stationary growth phase was observed by immunoelectron microscopy. Furthermore, we also demonstrated that the localization of the catalase depends on the growth phase in accordance with the extent of the catalase activity of the cells.

## Materials and Methods

### Chemicals

Standard chemicals, which were purchased from Wako Pure Chemicals (Osaka, Japan) unless otherwise stated, were of the highest grade available and were used without further purification. Sinapinic acid was purchased from Sigma-Aldrich (St. Louis, MO, USA). Ampholine and the p*I* marker, which were used for isoelectric focusing, were purchased from GE Healthcare (Buckinghamshire, UK) and Oriental Yeast (Tokyo, Japan), respectively. The molecular weight standard used for sodium dodecyl sulfate -polyacrylamide gel electrophoresis (SDS-PAGE) was purchased from APRO Life Science Institute (Tokushima, Japan).

### Bacterial Strain

*E*. *oxidotolerans* T-2-2^T^ was cultivated aerobically at 27°C in PYS-3 (peptone-yeast extract-saline) broth (pH 7.5) containing (per liter of distilled water) 8.0 g polypeptone (Nihon Pharmaceuticals), 3.0 g yeast extract (Kyokuto), 5.0 g NaCl and 5.0 g sodium succinate. The cell suspension was adjusted to OD_650_ = 0.5 and inoculated as one part per thousand to the culture medium. The standard culture condition was 500 ml of broth at 120 rpm. The inoculated medium was incubated for 18–20 h for the purification of the intracellular catalase and for 48 h for that of the extracellular catalase.

### Enzyme Assay Conditions

Catalase activity was measured spectrophotometrically by monitoring the initial decrease in absorbance at 240 nm caused by the disappearance of H_2_O_2_ per unit of time (min), using a spectrophotometer (Hitachi U-3210) at 25°C. Kinetic parameters, *V*_max_ and *K*_m_, were determined at 40°C. The H_2_O_2_ concentration was determined on the basis of the extinction coefficient of 43.6 M^−1^·cm^−1^ [25]. The standard reaction mixture for the assay contained 50 mM potassium phosphate buffer (pH 7.0), 30 mM H_2_O_2_ and 10 µl of catalase solution in a total volume of 1.0 ml. One unit of catalase activity (U) was defined as the amount of activity that decomposed 1 µmol of H_2_O_2_ per min. The enzyme activities are expressed as the means of at least four independent measurements.

### Purification of Catalase from Strain T-2-2^T^ (EktA)

All the purification steps were performed at 4°C. The spent medium obtained by centrifugation at 13,000×*g* for 15 min was applied onto Q-Sepharose Fast Flow (GE Healthcare; 2.5 cm×10 cm) equilibrated with buffer A (pH 8.0) consisting of 10 mM Tris-HCl and 1 mM 2Na-EDTA. The column was washed with 10 column volumes of buffer A and eluted with a linear gradient of 0−0.5 M NaCl-containing buffer A at a flow rate of 1 ml·min^−1^. The eluted fractions exhibiting catalase activity were concentrated and loaded on a Sephacryl S-300 (2.6 cm×100 cm), which had been equilibrated with 50 mM phosphate buffer containing 0.5 M NaCl at a flow rate of 0.25 ml·min^−1^. The eluted active fractions with high purity determined from the absorbance ratio of A_408_/A_280_ were collected. Thus, purified catalase was used as purified extracellular catalase.

The collected cells of strain T-2-2^T^ were suspended in buffer A (three times the weight of the cells). Then, 1 µg·ml^−1^ of DNase I (2000 U, Sigma-Aldrich) and 7 mM MgCl_2_ • 6H_2_O were added to the suspension, which was passed through a French pressure cell (SLM-AMINCO, Rochester, NY, USA) at 105 kgf/cm^2^. The resulting fluid was centrifuged at 13,000×*g* for 15 min to remove unbroken cells, dialyzed against buffer A and applied onto Q-Sepharose Fast Flow (2.5 cm×10 cm) equilibrated with buffer A (pH 8.0) consisting of 10 mM Tris-HCl and 1 mM 2Na-EDTA. The column was washed with 10 column volumes of buffer A and eluted with 10 column volumes of a linear gradient of 0−0.5 M NaCl-containing buffer A at a flow rate of 1 ml·min^−1^. The eluted fractions exhibiting catalase activity were concentrated and applied to a phenyl-Sepharose high-performance column (GE Healthcare; 1.6 cm×10 cm) equilibrated with buffer A containing 1 M ammonium sulfate. The enzyme was eluted with a linear gradient of 1.0−0 M ammonium sulfate in buffer A at a flow rate of 0.25 ml·min^−1^. The eluted fraction was concentrated and applied to a Sephacryl S-300 (2.6 cm×100 cm), which had been equilibrated with 50 mM phosphate buffer containing 0.5 M NaCl at a flow rate of 0.25 ml·min^−1^. The eluted catalase fraction was loaded onto Sephacryl S-300 again under the same condition. Thus, the purified catalase was used as purified intracellular catalase.

### Analytical Methods

The molecular mass of catalase treated with SDS was determined by SDS-PAGE on 12.5% (w/v) acrylamide gel (c-PAGEL, Atto, Tokyo, Japan) according to the method of Laemmli and Favre [26]. Isoelectric focusing in the disc gel was carried out in the presence of 2% (w/v) ampholine in the pH range from 3.5 to 10 using the purified intracellular and extracellular catalases. The molecular masses of the purified intracellular and extracellular catalases were determined by MALDI-TOF/MS (Voyager DESTR, Applied Biosystems). Sinapinic acid (saturated solution in acetonitrile/water [50∶50 v/v]) containing 0.1% TFA was used as a matrix. The sample was mixed with an equal volume of the matrix, 1 µl of which was spotted onto one well on the sample plate and then crystallized under an airflow. The protoheme content was determined by the pyridine ferrohemochrome method; 8% pyridine, 0.17 N NaOH and a small amount of Na_2_S_2_O_4_ were added to the catalase solution, and the heme content was calculated using millimolar extinction coefficient of Δε_557–580_ = 29 mM^−1^·cm^−1^ [27]. Bovine serum albumin dissolved in an equal volume of a mixture of milli-Q water and acetonitrile was used as the standard. Protein content was determined using a BCA protein assay reagent kit (Pierce, Rockford, IL, USA) with bovine serum albumin as the standard [28].

### Protein Sequencing

The purified extracellular catalase after the final purification step of passing through Sephacryl S-300 was separated by SDS-PAGE. The separated catalase band was transferred to a polyvinylidene fluoride (PVDF) membrane using a semidry blotter (AE-6677G Holize Blot). The transferred catalase band was excised from the membrane and applied to a protein sequencer (Model 491, Perkin-Elmer, Winter Street, MA, USA) to determine the N-terminal amino acid sequence of the polypeptide by Edman degradation [29].

### Effects of pH and Temperature on Catalase Activity

The effect of pH on catalase activity was assayed under standard conditions as described above except that the reaction mixture contained one of the following buffers (50 mM): pH 3.0−6.0, citrate-NaOH; pH 4.0−5.0, acetate-NaOH; pH 6.0−8.0, potassium phosphate; pH 8.0−9.0, Tris-HCl or pH 9.0−10.0, borate-NaOH.

The effect of temperature on catalase activity was assayed under standard conditions as described above except that the temperature of the reaction mixture was maintained at 5−80°C using a temperature-controlled cell holder in a spectrophotometer.

### Stability Studies

For determining the enzyme stability to H_2_O_2_, 1 ml of catalase (1000 U·ml^−1^) in a 1-ml dialysis tube was dialyzed against 1000 ml of buffer A containing 1 mM H_2_O_2_ at 30°C. After the incubation started, 100 µl of catalase solution was taken and immediately chilled on ice at 10 min intervals. Remaining activity was assayed under standard conditions as described above.

For determining the thermal stability of catalase, the enzyme (1000 U·ml^−1^) was incubated at different temperatures (25°C, 30°C, 35°C, 40°C, 45°C, 50°C, 55°C, 60°C, 65°C and 70°C) for 15 min. After incubation, the enzyme solution was immediately chilled on ice. Remaining activity was assayed under standard conditions as described above.

### Search for Catalase Genes in Genome DNA

DNA of *E*. *oxidotolerans* T-2-2^T^ was extracted from the cells using an ISOPLANT II kit (Nippon Gene, Tokyo, Japan) following the manufacturer’s instruction. To amplify a partial catalase gene sequence library from the DNA extracted from *E*. *oxidotolerans* T-2-2^T^, PCR was performed using the primer sets P1 (5′-AARAARYTIACIAAYCARGG-3′) and P2 (5′-CKIGCRTGIACIAYICKYTC-3′). The reaction mixture was subjected to PCR under the following conditions: 94°C for 2 min, 50°C for 30 s and 72°C for 1 min followed by 23 cycles at 94°C for 30 s, 50°C for 30 s and 72°C for 2 min and then 94°C for 30 s, 50°C for 30 s and 72°C for 10 min. The amplified catalase genes were purified using a QIA quick PCR purification kit (Qiagen) and were cloned in *Escherichia coli* DH5α with the pT7Blue-2 Vector system (Novagen) using a ligation kit (Takara) according to the manufacturer’s instruction. Plasmids were extracted and purified from 14 randomly selected clones using a Quantum prep Plasmid Miniprep kit (Bio-Rad) and the DNA sequence was determined by the dideoxy chain termination method with a BigDye terminator cycle sequence kit (Applied Biosystems, Forster City, CA) and an automated DNA sequencer (ABI Prism 3100 Genetic Analyzer; Applied Biosystems). The determined sequence was assigned by alignments using the GENETYX program (Genetec).

### Catalase Activity of Cell Suspension

Cells were harvested from the culture broth by centrifugation at 13,000×*g* for 15 min. The obtained cells were suspended in 10 mM Tris-HCl (pH 8.3) in the same volume of the supernatant. To examine the catalase activity of the cell extract, the cell suspension was applied to a multibeads shocker (Yasui-Kikai, Osaka, Japan) at 2500 rpm for 6 min to disrupt the cells. The catalase activity was examined as described above for the adjustment of the total catalase activity of the cell suspension based on that of the cell extract. The prepared cell suspension was reacted with 25–80 mM H_2_O_2_ and an aliquot of the sample was obtained from the reaction mixture in a time-dependent manner. The H_2_O_2_ concentration in the obtained reaction mixture was determined on the basis of previous reports [30], [31] as follows: 0.4 ml of the sample solution was added to 0.4 ml of 5% Ti(SO_4_)_2_, and its absorbance at 408 nm was determined.

### Immunolocalization of Catalase

Cells were obtained by centrifugation from 5, 14 and 24 h cultures, which correspond to mid-exponential, early stationary and mid-stationary growth phases, respectively, at 27°C in PYS-3 as described above. Preparation of rabbit-EktA antiserum was performed by Takara. Obtained cells were fixed with 5% paraformaldehyde in PIPES buffer (pH 7.2). These cells were dehydrated through a graded series of ethanol concentrations and embedded in L-R White resin (London Resin), which was polymerized in gelatin capsules at 4°C under UV light for 2 days. The ultrathin sections, which were obtained using a diamond-knife-equipped ultramicrotome, were transferred to 200-mesh nickel grids for subsequent immunolabeling at room temperature. Each rabbit-anti-EktA antiserum and a 10-nm-diameter gold-particle-conjugated secondary antibody (anti-rabbit IgG, Goat, Po, Gold, GAR10, Funakoshi) were diluted 1∶160 in 100 mM phosphate buffer (pH 7.0). The grids were blocked by adding a drop of 100 mM phosphate buffer (pH 7.0) containing 1% bovine serum albumin, followed by rinsing with 100 mM phosphate buffer. They were then labeled with the primary antibody overnight and with the secondary antibody for 2 h at 4°C. Unbound antibodies were washed off by sequentially floating the grids on a drop of phosphate buffer and distilled water. After the labeling, the grids were dried and stained with 2% (w/v) aqueous uranyl acetate and Sato’s Pb solution [32]. The preparation was observed with a transmission electron microscope (model H-800; Hitachi).

## Results

### Comparison between Intracellular and Extracellular Catalases

The production of extracellular catalase by strain T-2-2^T^ and the similarity between intracellular and extracellular catalases have been reported [19]. However, the authors provide no direct evidence that intracellular catalase is secreted. To clarify whether the extracellular catalase exhibits the same molecular features as those of the intracellular catalase, the characteristics of both catalases were studied in more detail.

#### i) Molecular weight and isoelectric point

The molecular weights of the extracellular and intracellular catalases were determined by MALDI-TOF-MS. In addition, the molecular weight of the mixture of extracellular and intracellular catalases was also determined. The molecular masses of the extracellular and intracellular catalases and the mixture were 56,516±102, 56,448±56 and 56,481±54 Da, respectively. The molecular mass according to the amino acid sequence of this catalase was calculated to be 56,488 Da. From the above results, although the molecular mass of the intracellular catalase is approximately 70 Da smaller than that of the extracellular catalase, these catalases are identical, based on the calculation of the significant difference by Student’s *t*-test and a *p* value of 0.05.

The isoelectric points (p*I*) of the extracellular and intracellular catalases and the mixture were determined. All the samples exhibited the same p*I* of 3.7. This value is much smaller than the p*I* predicted on the basis of the amino acid sequence of EktA.

#### ii) N-Terminal sequence

The N-terminal sequence of the extracellular catalase was determined using an amino acid sequencer. The determined sequence was “MNENEKKLTTNQGVPIXDNQ”. This sequence is identical to that of the previously reported EktA except for the undetermined 17th residue [30]. Therefore, it is considered that there is no modification or cutting of the N-terminal sequence of the extracellular catalase in comparison with that of the intracellular catalase.

#### iii) Enzymatic characteristics

**Figure 1:**
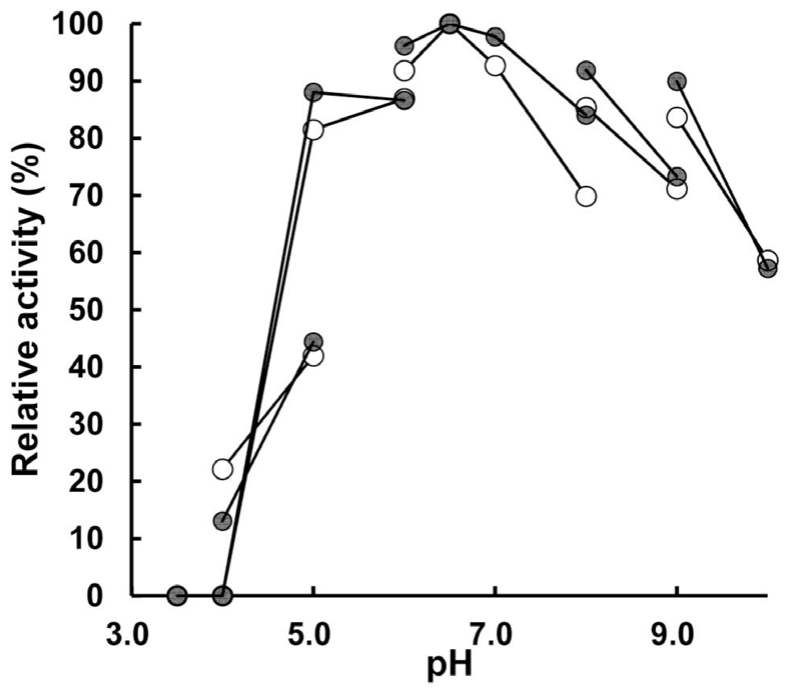
Effect of pH on activities of extracellular and intracellular catalases of *E. oxidotolerans* T-2-2^T^. Extracellular and intracellular catalases are indicated by filled and open circles, respectively. The buffers (50 mM) used were as follows: pHs 3.0–6.0, citrate-NaOH; pHs 4.0–5.0, acetate-NaOH; pHs 6.0–8.0, sodium phosphate; pHs 8.0–9.0, Tris-HCl; pHs 9.0–10.0, borate-NaOH. The activities are relative to that at pH 6.5, which is 100%.

The pH and temperature dependences of catalase activity were determined (Fig. 1, 2[a]). Both the extracellular and intracellular catalases exhibited similar temperature-dependent activities with a clear decrease in activity at temperatures above 70°C. This temperature dependence was not observed in *Micrococcus luteus* catalase. This reflects the fact that strain T-2-2^T^ was isolated from a cold environment. In terms of the pH dependence of catalase activity, both catalases exhibited optimum activities at approximately pH 6.5, and the activities were relatively high (relative activities: 57–100%) between pHs 5 and 10 compared with those at pH below 5. In conclusion, there are no marked differences in the temperature and pH dependences between the extracellular and intracellular catalases.

**Figure 2:**
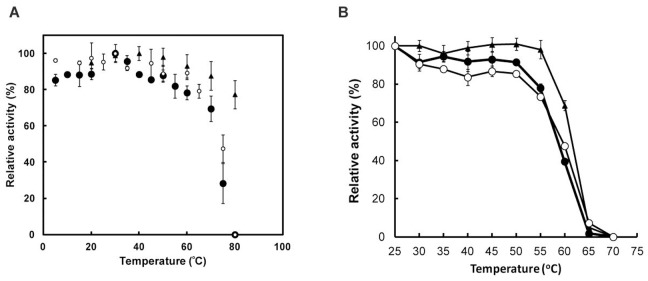
Effects of temperature on activities and stabilities of extracellular and intracellular catalases of *E. oxidotolerans* T-2-2^T^. (A) Effect of temperature on catalase activity. Extracellular and intracellular catalases are indicated by filled and open circles, respectively. *M*. *luteus* catalase, indicated by closed triangles, was used as a counterpart mesophilic enzyme. Catalase activity was assayed, as described in Materials and Methods, at the temperature indicated. (B) Temperature stability of catalase. Symbols are indicated as in (A). The enzyme was incubated for 15 min at the indicated temperatures prior to activity measurement. Catalase activity was assayed at 25°C as described in Materials and Methods.

Kinetic parameters, *V*_max_ and *K*_m_, were determined. Both the extracellular and intracellular catalases exhibited the same *V*_max_ and *K*_m_ values of 1.5×10^6 ^µmol H_2_O_2_ µmol·heme^−1^·s^−1^ and 40 mM, respectively. The *V*_max_ value was higher than those of *M*. *luteus* (6.4×10^5 ^µmol H_2_O_2_ µmol·heme^−1^·s^−1^) and bovine liver (2.2×10^5 ^µmol H_2_O_2_ µmol·heme^−1^·s^−1^) catalases. The high *V*_max_ value of EktA reflects the catalase is isolated from hydrogen-peroxide-tolerant microorganism.

The effects of temperature and H_2_O_2_ on the stabilities of the extracellular and intracellular catalases were also examined. Both catalases were relatively stable up to approximately 50°C and their activities began to decrease above 55°C (Fig. 2[b]). The temperature stabilities were equivalent to that of *M*. *luteus* catalase. Surprisingly, this characteristic is different from that of the ordinary cold-adapted enzyme [17], [33], [34]. The hydrogen-peroxide-tolerant nature attributed to the environmental pressure that the microorganism overcomes may account for the robustness of the enzyme. In addition, these catalases were stable against 1 mM H_2_O_2_ over a 1 h incubation period (data not shown). On the basis of the above results, there is no difference in enzymatic features between the extracellular and intracellular catalases.

On the basis of the molecular features and enzymatic properties of the extracellular and intracellular catalases, it can be concluded that both catalases are the same molecule. Therefore, it is predicted that catalase is secreted extracellularly via the transport of intracellular catalase. However, there is no signal peptide sequence upstream of *EktA*. The mechanisms by which the intracellular catalase is transported into the extracellular space are unknown.

### Search for Catalase Genes in Genome DNA

In the search for sequences of catalase genes, gene amplification was performed by PCR using primer sets designed on the basis of the sequence of *EktA*. The amplified gene products (158 bp) were ligated to the T-vector and then transformed into competent cells of *E*. *coli*. The determined sequences were ascribed to two almost identical catalase gene sequences, clade 1 catalase (7 clones; deduced amino acid sequence: “VPIGDNQNSRTAGRRGPTLLEDYQLIEKIAHFDRERV”), which corresponds to the catalase of the present study, and clade 2 catalase (7 clones; deduced amino acid sequence: “LKMAEDEFSLKAGLRGPTLIEDFHFREKMTHFDHERI”), by the determination of the gene sequence of 14 clones. The results suggest that strain T-2-2^T^ possesses only one clade 1 catalase gene. The complete genome sequence of a strain, *Exiguobacterium* sp. AT1b, belonging to the same genus as that of strain T-2-2^T^, indicates that the former possesses clade 1 and clade 2 catalases [35].

### Catalase Activity of Cells Depending on Growth Phase

**Figure 3:**
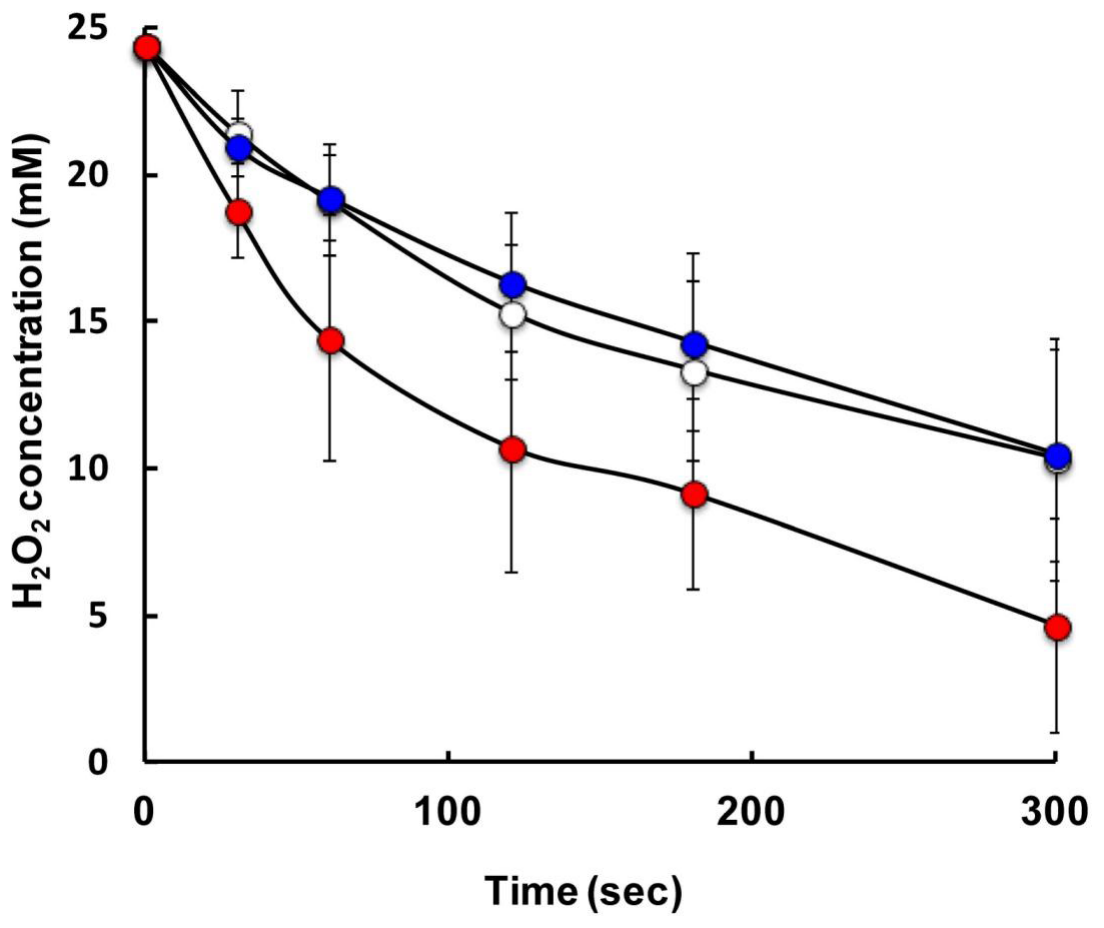
Degradation of hydrogen peroxide in cell suspension of *E. oxidotolerans* T-2-2^T^ obtained from 5, 14 and 24 h cultures. Culture periods of 5, 14 and 24

Comparison of the catalase activity of the cells depending on the growth phase was performed by adjusting the total catalase activity of the cell suspension based on that of the cell extract. Therefore, the difference in the catalase activity will reflect the localization of the catalase in the cells. If many of the catalase molecules locate on the surface of the cells, the catalase activity of the cell suspension is expected to be high. Cell suspensions from different growth periods, 5, 14 and 24 h of culture, which correspond to exponential, early stationary and mid-stationary growth phases, were prepared. While cell suspensions from 5 and 14 h cultures exhibited similar catalase activities, the cell suspension from the 24 h culture exhibited higher activity (Fig. 3). This indicates that catalase localization is more efficient in the cells from the 24 h culture than those from the other cultures. Catalase activity (U) per cell density (OD_650_) for each culture period was also estimated. This result showed that cells from the 24 h culture exhibited a much higher activity (24,000 U/ml/OD_650_) than those from the 5 (4700 U/ml/OD_650_) and 14 (3100 U/ml/OD_650_) h cultures. The results described above indicate that cells in the mid-stationary growth phase exhibit efficient localization of catalase for the discrimination of H_2_O_2_ as well as higher catalase content than the cells in other growth phases.

### Immunolocalization of EktA

**Figure 4:**
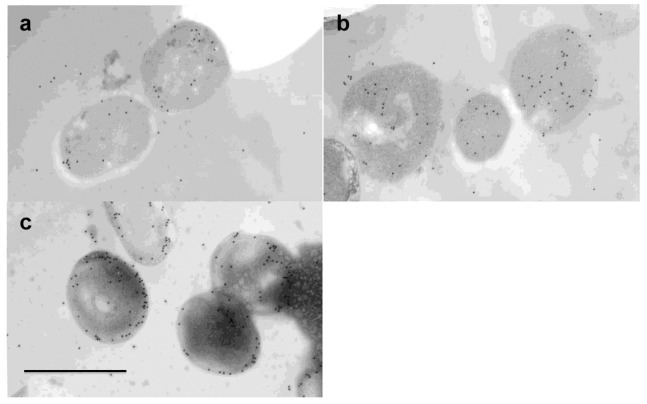
An ultrathin section showing immunolocalization of catalase in *E. oxidotolerans* T-2-2^T^ after 5, 14 and 24 h of culture. The particles show the localization of catalase. Cells from 5, 14 and 24-exponential, early stationary and mid-stationary growth phases, are shown in (a), (b) and (c), respectively. Bar, 1 µm.

**Figure 5:**
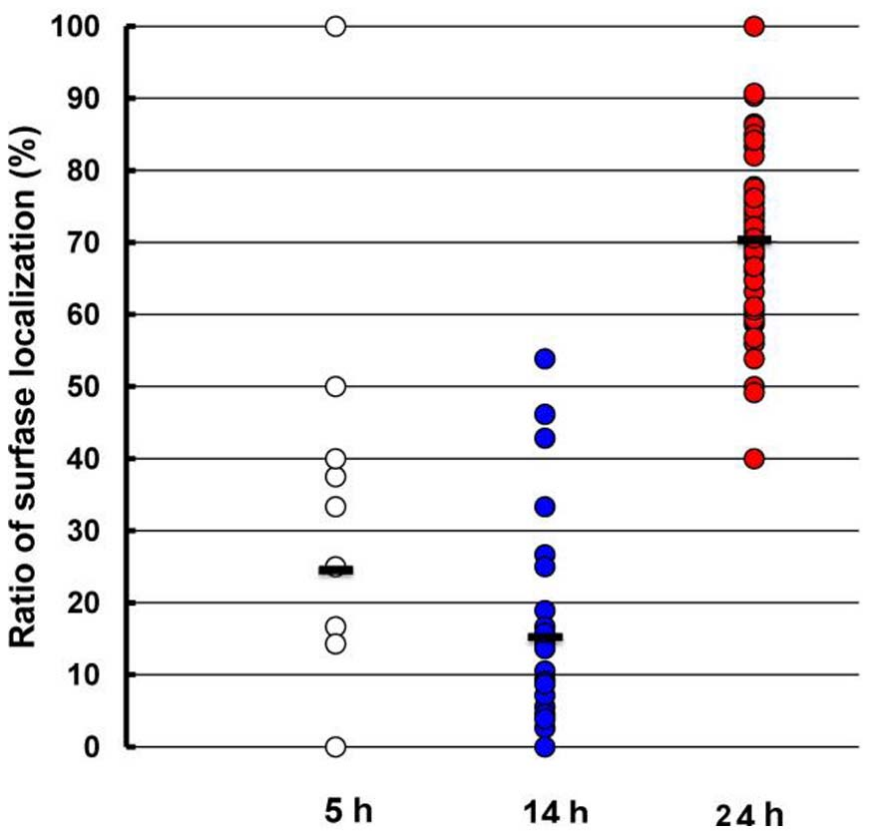
Inner circumference localization ratio of catalase in strain T-2-2^T^ cells on the basis of particles in ultrathin sections exhibiting catalase immunolocalization. Cells from 5 (n = 19), 14 (n = 33) and 24 (n = 47) h cultures are indicated by open, blue filled and red filled circles, respectively. Particles locating within 50 nm from the surface of the cell are considered as surface- localized particles. Black bars indicate the average inner circumference localization ratio in each growth phase.

It was considered that the cellular localization of catalase is an important factor for the efficient discrimination of extracellular H_2_O_2_. The cellular localization of EktA in strain T-2-2^T^ was estimated by immunoelectron microscopy using anti-EktA antiserum and a gold-particle-conjugated secondary antibody. Figure 4 shows the localization of EktA-detecting particles in strain T-2-2^T^ in mid-exponential (5 h culture) to mid-stationary (24 h culture) growth phases. Although the specific localization of catalase was not observed in the cells in both the mid-exponential and early stationary growth phases, the localization of catalase in the inner circumference was observed in the cells in the mid-stationary growth phase. In addition, the inner circumference structure of the cells in the mid-stationary growth phase was different from those of the other cells. The inner circumference localization ratio of catalase in strain T-2-2^T^ cells on the basis of particles in ultrathin sections exhibiting catalase immunolocalization was estimated (Fig. 5). This result also indicated high catalase localization in the inner circumference of the cells in the mid-stationary growth phase. The average inner circumference localization ratios of catalase in the mid-exponential, early stationary and mid-stationary growth phases were 27%, 14% and 71%, respectively. The results described above confirmed the high catalase activity of the cell suspension prepared from the cells in the mid-stationary growth phase.

### Effect of Detergent (Tween 60) Treatment on Catalase Activity of Cells

**Figure 6:**
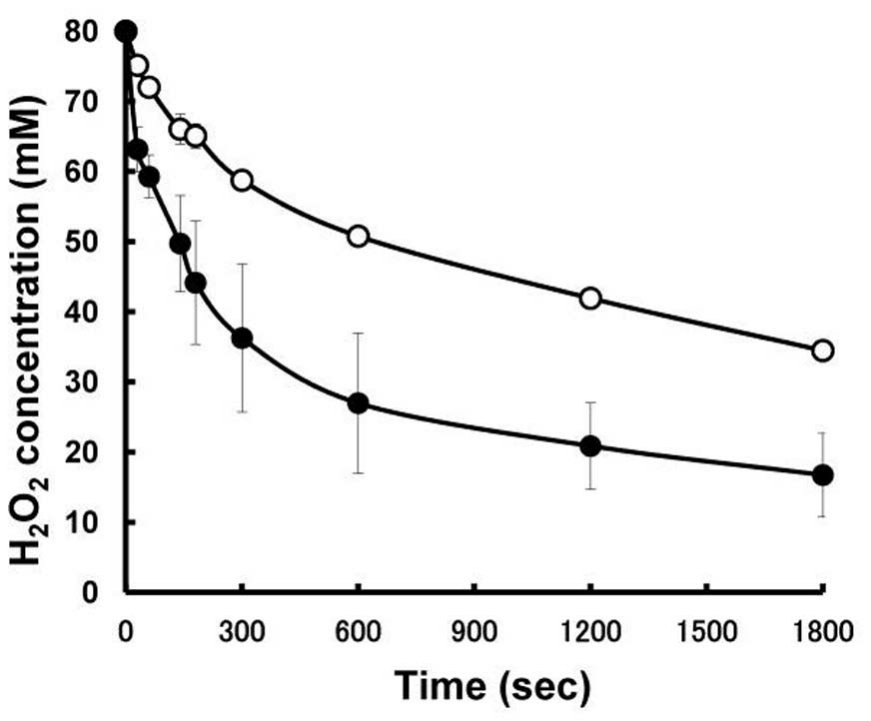
Degradation of hydrogen peroxide in cell suspension of *E. oxidotolerans* T-2-2^T^ and that in 0.1% Tween 60-treated cell suspension. The untreated cells and 0.1% Tween 60-treated cells are indicated by open circles and filled circles, respectively. The cells were obtained after 36 h of cultivation. The amount of the cells was adjusted as OD_650_ = 0.005 in the final reaction mixture. The reaction was performed at 25°C.

The effect of detergent treatment on the catalase activity of the cells was estimated. To ascertain that the addition of 0.1% Tween 60 does not induce cell disruption, viable cell counts were carried out in detergent-treated and untreated cultured broths. The viable cell count of the untreated culture was 7.0×10^9^ (CFU/ml), while that of the detergent-treated culture was 6.2×10^9^ (CFU/ml). Therefore, it is considered that the detergent treatment scarcely affected the viability of strain T-2-2^T^ cells. The catalase activity of Tween 60-treated cells decreased in comparison with that of the untreated cells (Fig. 6). At the same time, the catalase activity of the supernatant of the detergent-treated culture broth increased in comparison with that of the untreated control (data not shown). Hence, the obtained results demonstrated that catalase molecules found in the vicinity of the cell surface can be removed by detergent treatment without cell disruption. This result suggests that the catalase of strain T-2-2^T^ is not strongly fixed in the vicinity of the cell surface.

## Discussion

Although catalase can react with H_2_O_2_ inside the cells, it is better to eliminate H_2_O_2_ before it enters the intracellular space. In this study, we demonstrated that catalase localized in the inner circumference of strain T-2-2^T^ cells. It is considered that the bacterium exhibits strong H_2_O_2_-eliminating activity owing to this catalase localization. In this state, the individual cell itself exhibits catalase activity. A similar case of catalase localization near the cell surface was observed in the following Gram-negative bacteria: *Vibro rumoiensis*, which possesses catalase on the cell surface as well as in the periplasmic space [12]–[14] and *Vibrio fisheri*, which possesses catalase in the periplasmic space that eliminates H_2_O_2_ produced by the host squid in order to survive in the light organ of the host [11]. On the other hand, a member of Gram-positive *Actinobacteria*, *Mycobacterium tuberculosis*, which possesses catalase and superoxide dismutase in the thick capsule, is pathogenic to humans [36]. In the case of strain T-2-2^T^ cells, the periplasmic space is not present as a reservoir of catalase molecules in the vicinity of the cell surface. Instead, the peptidoglycan or S-layer is a candidate catalase reservoir. Many Gram-positive Firmicutes possess the S-layer, which is 3–35 nm thick, outside the peptidoglycan layer [37]. It is known that the S-layer can attach proteins [37]–[39], which exhibit a noncovalent binding. In the case of strain T-2-2^T^, the surface layer was observed in the cells in the mid-stationary growth phase and the catalase was loosely bound to the cells. Therefore, it is likely that strain T-2-2^T^ reserves its catalase as an S-layer-attached protein as a defense against extracellular H_2_O_2_. On the other hand, it has been reported that cell-wall-associated proteins are tightly bound to the peptidoglycan layer by covalent binding [40].

To ascertain that the extracellular catalase is the same molecule as the intracellular catalase, the molecular features and enzymatic characteristics of these catalases were examined. From the results, it was concluded that *E*. *oxidotolerans* T-2-2^T^ releases the same intracellular catalase molecule extracellularly into the culture medium. There are two types of transport system across the membrane, Sec and Tat [41]. The Sec transport system secretes unfolded proteins. On the other hand, the Tat system secretes folded proteins. *Escherichia coli* produces hydrogenase-2 which consists an Fe-S cluster-binding subunit (HybO) and Ni-Fe active site cofactor (HybC). It has been reported on Tat dependent transport of HybOC [42]. It is expected that it is difficult to secrete unfolded catalase because the molecule consists of a subunit structure that includes a prosthetic group, heme. In addition, there is no signal peptide sequence upstream of the catalase gene sequence and the bacterium has no periplasmic space. Therefore, it is more likely that the catalase is secreted via the Tat system. It has been reported that KatA of *Helicobacter pylori* is secreted via the Tat system [43]. Although the signal peptide sequence of the Tat system does not exist upstream of *KatA*, the sequence is connected to the *KapA* sequence, which is located in the same gene cluster as *KatA*. The signal peptide sequence of the Tat system in the determined gene sequence around the *EktA* was not detected. It is suggested that the EktA catalase is involved in a secretory pathway independent of the signal peptide. Bendtsen *et al*. (2005) developed a method of predicting the identity of proteins following a signal-peptide-independent secretory pathway according to the properties of cellular proteins such as amino acid composition, secondary structure and disordered regions [44]. According to the method, it can be predicted that proteins exhibiting a SecP score higher than 0.5 are secretory proteins. The SecP score of EktA was determined to be 0.62. Therefore, it can be predicted that EktA is a secretory protein.

Protein secretion and surface display in Gram-positive bacteria have been explained on the basis of a signal-peptide-bearing base. However, the catalase gene of strain T-2-2^T^, *EktA*, does not have a signal peptide. Therefore, there is a possibility that the surface display system of the catalase of strain T-2-2^T^ involves a novel mechanism. The inner circumference of the cells was observed only in the mid-stationary growth phase. This may suggest that this defense mechanism is more required in the low metabolic state than in the state of vigorous metabolism. Although strain T-2-2^T^ secreted catalase, it has not been understood if the catalase molecules are trapped during their secretion or the catalase molecules excreted into the outside of the cells are trapped at the inner circumference of the cells. We do not know if the surface display mechanism of catalase in strain T-2-2^T^ is common within the same genus. *Exiguobacterium* spp. are widely distributed in extreme environments such as Antarctica and in the permafrost of polar regions [45]. Microorganisms present in cold and desiccated environments may be in a low metabolic state during a long period under low metabolic activity. The low temperatures induce loss of catalase activity in *Vibrio vulnificans* [46] and desiccation causes a significant decrease in the catalase activity in mosses [47]. There is a possibility that the surface display mechanism of catalase is common to *Exiguobacterium* spp. that survive in extreme environments.
